# Facts and Philosophy of Dental Progress, No. 2

**Published:** 1867-07

**Authors:** 


THE
AMERICAN JOURNAL
OF
DENTAL SCIENCE.

Vol. I. THIRD SERIES.–JULY, 1867. No. 3

### ORIGINAL COMMUNICATIONS.

### ARTICLE I.

### *Facts and Philosophy of Dental Progress, No. 2.*

By Professor AUSTEN.

PROGRESS in some direction is an essential condition of thought and action. In the individual its extent is necessarily limited : in art and science it is illimitable The place reached by the man of science is determined by his starting point, rate of progress and duration of life : but the science itself moves on from age to age and can only cease to grow, when all truth shall have been found out. When the finite mind can grasp the infinite, progress may end: until then we are like Newton, children handling pebbles on the shore of a boundless and fathomless ocean.

No art or science, dependent for its progress upon the exercise of observation, reason and skill, can truly be said at any time to be perfect—that is, incapable of further improvement. Yet we are constantly met with the statement, that this or that act or invention has been brought to a "state of absolute perfection." Some improvements are so great and rapid, and some discoveries are so astounding, that the mass of men may be perhaps excused for thinking that further progress in that direction is impossible. But to the truly scientific, this wide space between the past and present gives the stronger presumption of a still wider unexplored interval between the present and future.

In arts which advance with such rapidity as dentistry, some will fall into this error and remaining content with the present, seem not to contribute their quota to progress. Others commit the greater error of constantly seeking after something new; neither acknowledging the value of past discovery, nor willing to test properly the discoveries of the present. The latter, rejoicing in the soubriquet of *Young America*, speak contemptuously of the former as *Old Fogeys*: not knowing that old fogeyism is the balance wheel, which gives steadiness of motion to their spasmodic impulses. In subsequent papers frequent instances will be given, where this conservative element has proved most valuable to dentistry. At no time is its influence demanded more than now, when so many practitioners seek to enlarge their business, less by doing an old thing well, than by always offering the "latest thing out."

Our *quarterlies* are replaced by *monthlies* for fear some new discovery should have a month's trial before its general adoption. Even the American Journal of Dental Science offers, in sacrifice to the insatiate craving for novelty, its dignity as a quarterly. This is *not* progress: the spirit of Young America is not progress. When our last quarterly expires for want of an "old fogey" subscriber, we may begin to date the decline of dentistry.

When I speak, therefore of the rapid progress of dentistry, and seek to learn its causes, I do not refer to that restless search after what is new, which misleads more than it benefits: but to those well-tried principles, methods and materials, which have added ten-fold to the power which is wielded by the skilful dentist; still more to the generous spirit which understands that in giving lies the true secret of receiving; also to an increasing acknowledgement of the truth that collateral study is essential to a knowledge of any speciality.

FIRST, among the causes of the rapid progress of dentistry, I name EDUCATION. Men cannot improve what they do not understand; cannot understand what they do not study; and cannot study without opportunity and a certain degree of mental and physical aptitude. Education, in exact proportion to its completeness, increases the mental power, improves manual skill and gives the opportunity: cultivates habits of study and greatly enlarges the understanding. Thus education gives a man knowledge of the present status of his profession, teaches him its wants and strengthens those faculties, which he may use for its improvement.

A constant result of defective professional education is the waste of time and talent in overcoming difficulties already mastered. Hence, other things equal, that system of instruction is most perfect, which gives the most complete training and acquaints the student most thoroughly with what has already been done in his profession. Text books, journals, societies, colleges and office tuition, are the principal instruments of education. When dental journals, text-books, colleges and societies had no existence, office tuition was a very imperfect teacher: imparting doubtless much valuable instruction, but limited more or less to the experience of one man. It can now be made far more effective, because of facilities enjoyed by the teacher himself, for uniting with his student in profiting by the ideas and discoveries of others.

An association of teachers, having education as their chief aim, *should* be able to teach more effectively than the single practitioner, whose first concern is his practice, often so engrossing as to leave little time for much else. A college (or collection) of teachers, organized upon correct principles and faithful to their duty, may be regarded therefore, without disparagement to the system of office teaching which has always existed, as an important advance in education, and hence a powerful agent in dental progress. But the five instruments of education are in truth inseparable and should work together. If I seem here to compare them, it is not to argue that some one is to be preferred, or some other excluded: but rather to show that dental education has largely added to and improved its instruments, and thus to account in part for that rapidity of progress, which is the present subject of inquiry.

The SECOND cause I shall name is at once a consequence of, and an aid to, education. I mean that spirit of liberality which must necessarily mark any body of men claiming to belong to one of the "liberal professions." So long as the spirit of secrecy, once universal, is practiced by any considerable number, progress is slow. The student may improve upon his preceptor's ideas and give in turn to his own students the benefit of a double experience. They may, by the aid of well-locked laboratories and better locked mouths, manage for a long time to reap the sole benefit of some valuable discovery. But what is the gain of the very best invention, compared with the immense loss which such professional selfishness, if universal, would entail. No where is the truth more forcibly shown than in Art and Science, that the freest giver is ever the largest receiver: for no single experience can prove as valuable as the united contributions of many minds.

The great truths of science are mastered by no one person, nor in any single age. Newton made use of the labors of Kepler and Copernicus: and succeeding minds, less great, have modified his theories and improved his inventions. It is thus that Art grows, each age beginning where the former left off; but under the rule of secrecy every period must work out first principles for itself and progress becomes impossible.

The old philosophers asserted that "Nature abhors a vacuum." Modern philosophers know that *science* abhors a *secret*. Science is knowledge, a secret is unknown: between them there is an "irrepressible conflict." Hence the opprobrium resting upon the quack nostrum, possibly very good in itself. Secrecy is the essence of quackery, and the secret-keeping dentist is a charlatan, who in this age of "patents" is utterly without excuse for refusing to contribute his share towards the progress of his Art. Societies, journals and colleges have given great impetus to dentistry, by liberalizing the profession, and encouraging the free interchange of ideas.

The man who gratuitously imparts his discovery has a truly scientific unselfishness, and a catholic spirit worthy of high praise. We might wish all to do this, but can scarcely expect it. Nor, so long as men of science copyright the books in which they give to the world the result of their investigations, can they consistently object to the principle of *patent*-rights. Dentists at the present day are much annoyed with the multiplicity of patents and their management. There are many worthless patents and many dishonorable patentees, but the *right* to patent an invention is undeniable, and it *may* be done in a manner consistent with the interest of science. That it is not so done in dentistry is the fault, in part of the patentee, in part of the profession.

The originator of an invention or discovery is entitled to his reward. If he is content with the honor, the profession will not object to the form of payment. If he seeks a more substantial return, it becomes a matter of barter and subject, of course, to all the be-littling influences of trade. So long as members of any profession use ideas without acknowledgment; so long as they derive pecuniary benefit from inventions, and yet refuse pecuniary return to the inventor, so long will patents be a source of contention, materially injuring the *moral* status of that profession. But the injury to science and art is in one respect slight, because the *secret* is made "patent," and thus progress is not arrested.

The THIRD and last cause of the rapidity of Dental progress which I shall notice is found in its peculiarly

MECHANICAL character, which permits it to be so largely benefitted by the inventive spirit of the age. A man may be a good physician, yet not know one tool from another; a dentist is a mechanic, or he is nothing. The former may loose his right arm, yet write prescriptions with his left; the latter must have two skillful hands, and know when and where to use them. The Surgeon may be a poor judge of painting and a worse critic in culpture, but the Dentist, who has no æsthetic faculty and is wanting in the ability to appreciate works of art, lacks a most important element of dental character.

Dentistry in its progress, benefits by all those discoveries in science which have done so much for medicine and surgery, but it has moved faster than they, by virtue of this artistic element, which is its distinguishing characteristic, as one of the healing arts. So far, then, from undervaluing this element, or keeping it in the back ground, as some very strangely seek to do, I would place before you MECHANICAL SKILL—skill in the use of instruments—as absolutely essential in every practical application of dental science. I would have you understand that, whilst you may and should possess knowledge and abilities which can make you good surgeons and physicians, unless you possess something more, you cannot become good dentists. I would not in this wish to be considered as placing dentistry above its sister arts, I simply state that it requires something which they do not—and it is that which constitutes the "*differentia*" of Dental Science.

These specific requirements involve physical and mental qualities far from common, hence and because of defective education, a large proportion of incompetent dentists. Hence so few, comparatively, attain eminence in this profession. Hence again, the great necessity for a judicious selection of students, and a careful examination of candidates for practice. For there are many whom no amount of training can qualify for the practical application of their art, however learned in its science.

In a series of papers on Dental Education, I propose to consider more at length the two first causes here assigned, of rapid progress in dentistry. In the remaining papers of this series, I shall confine the inquiry to the third and last cause—the mechanical or artistic element. I shall briefly review the several instances of progress, without, however, entering into a full description of the details of the various processes, for my object is not to write a practical treatise on mechanism: this must be left for another place. But I shall, I think, render you a good service, if I give you a correct view of the improvements in dental art.

I may, perchance, wound the conceit of Young America by telling him that he deserves no credit for doing some things better than his old preceptor. I may may vex him by advising a return to some relic of old fogeyism, I may even enrage him by intimating that in his haste to improve he has at times made worse, or venturing to hint that two years test of his pet practice is less convincing than twenty years test of a method he could not or would not learn. I shall hope, however, to give evidence of progress, sufficient to make every student or young practitioner rejoice that he is not called upon to battle with the difficulties and deficiencies of Dental Science as it once was.

